# Optical Study of the Electronic Structure and Lattice Dynamics of NdBaMn_2_O_6_ Single Crystals

**DOI:** 10.1038/s41598-019-54524-0

**Published:** 2019-12-03

**Authors:** Rea Divina Mero, Kirari Ogawa, Shigeki Yamada, Hsiang-Lin Liu

**Affiliations:** 1 Department of Physics, National Taiwan Normal University, Taipei, 11677 Taiwan; 2 Department of Material System Science, Yokohama City University, Yokohama, 236-0027 Japan

**Keywords:** Materials science, Condensed-matter physics

## Abstract

We investigated the electronic structure and lattice dynamics of double perovskite NdBaMn_2_O_6_ single crystals through spectroscopic ellipsometry and Raman scattering spectroscopy. The optical absorption band centered at approximately 0.88 eV was assigned to on-site *d*–*d* transitions in Mn, whereas the optical feature at approximately 4.10 eV was assigned to charge-transfer transitions between the 2*p* state of O and 3*d* state of Mn. Analysis of the temperature dependence of the *d-d* transition indicated anomalies at 290 and 235 K. The activated phonon mode, which appeared at approximately 440 cm^−1^ alongside with the enhancement of the 270 cm^−1^ phonon mode, coupled strongly to the metal–insulator transition at 290 K, which was associated with a charge/orbital ordering. Moreover, the MnO_6_ octahedral breathing mode at 610 cm^−1^ exhibited softening at a temperature lower than 235 K (temperature of the antiferromagnetic phase transition), which revealed the strong coupling between the lattice and magnetic degrees of freedom. The spin–phonon coupling constant obtained was *λ* = 2.5 cm^−1^. These findings highlight the importance of charge–orbital–spin interactions in establishing NdBaMn_2_O_6_ phases with novel properties.

## Introduction

Colossal magnetoresistance has promising applications. The search for room-temperature colossal magnetoresistance has resulted in the creation of new perovskite manganites^1–4^. Perovskite manganites have unique physical properties because of the interactions among the charge, orbital, and spin degrees of freedom^5–9^. Studies have been conducted extensively on perovskite manganites, which have the general formula *R*_1*−x*_*A*_*x*_MnO_3_ (*R*: rare-earth cation; *A*: alkaline-earth cation)^10–12^. The random distribution of *R*^*3+*^ and *A*^*2+*^ causes this system to have inherent disorder in the lattice^13^. This randomness makes it difficult to characterize the effect of lattice distortions on the physical properties. By contrast, double perovskite manganites have an *A*-site ordered structure that eliminates randomness^14–18^. Their general formula is *Re*BaMn_2_O_6_, where *Re* refers to rare-earth elements, such as Tb, Dy, Ho, Sm, Eu, Gd, La, and Nd. The structure of double perovskite manganites comprises alternately stacked layers of *Re*O, MnO_2_, and BaO along the *c*-axis. This creates a MnO_2_ square sublattice sandwiched between two types of rock-salt layers (*R*O and BaO) with substantially different sizes. Furthermore, *Re*BaMn_2_O_6_ can be classified into three categories according to its crystal structure and the mismatch between the sublattices. Small *Re* elements such as Tb, Dy, Ho, and Y are monoclinic, at room temperature and exhibit large octahedral tilting. *Re* elements such as Sm, Eu, and Gd are tetragonal and exhibit a marginal tilt in the MnO_6_ octahedra. La, Pr, and Nd also have a tetragonal structure; however, because of their relatively large ions, they exhibit no octahedral tilt at room temperature^19,20^. Generally, the mismatch between the trivalent *Re* and divalent Ba increases the Mn–O bond lengths on the *Re*O side and decreases them on the BaO side. Moreover, the Mn–O–Mn bond angles have a deviation of at least 5° for an angle lower than 180°. The changes in the bond lengths and bond angles decrease the bandwidth^19,21^.

*Re*BaMn_2_O_6_ has a complex phase diagram, and the phases of *Re*BaMn_2_O_6_ are classified into three groups. The first group has the largest mismatch and exhibits three transitions: a structural transition at high temperature, a charge/orbital-order transition associated with a metal–insulator transition, and an antiferromagnetic transition during cooling. The second group has an ionic radius smaller than Sm and exhibits a CE-type charge/orbital-ordered ground state, which is stable at high temperatures. Moreover, the second group does not exhibit a structural transition. The third group exhibits a ferromagnetic transition as well as a transition from the ferromagnetic state to the *A*-type antiferromagnetic state^19,21^. Generally, *Re*BaMn_2_O_6_ has a similar phase diagram with the *R*_1*−x*_*A*_*x*_MnO_3_ except that the former has higher charge/orbital-order transition which is stable around the element Nd. The structural transition without charge and magnetic ordering that occurs above the charge/orbital ordering temperature is also unique in *Re*BaMn_2_O_6_.

The properties of the third group of phases are interesting. The rare absence of octahedral tilt in these compounds at room temperature makes them ideal for understanding how it affects phase transitions. The properties of NdBaMn_2_O_6_ are also worth investigating because NdBaMn_2_O_6_ is located in a critical region between the phase boundaries of the charge/orbital-ordered, *A*-type antiferromagnetic, and ferromagnetic phases. A strong competition among phases, particularly between the ferromagnetic metal and antiferromagnetic insulator, is crucial for colossal magnetoresistance.

Previous studies have indicated that polycrystalline NdBaMn_2_O_6_ has a tetragonal crystal structure with the space group *P4/mmm* at 400 K^21–23^. NdBaMn_2_O_6_ exhibits a paramagnetic to ferromagnetic metal transition at 300 K, followed by a ferromagnetic to *A*-type antiferromagnetic metal transition at 290 K^22^. One study reported the ferromagnetic to antiferromagnetic transition and associated resistivity change to occur at 275 K^21^, whereas another reported its occurrence at 210 K^23^. The ferromagnetic to antiferromagnetic transition is associated with a change in the lattice parameters where the *ab*-plane expands and the *c*-axis shortens, which coincides with the charge/orbital ordering^21,24^. Furthermore, the transitions lead to interesting phenomena observed at approximately room temperature, which increase the functionality of NdBaMn_2_O_6_^23,25^.

**Figure 1:**
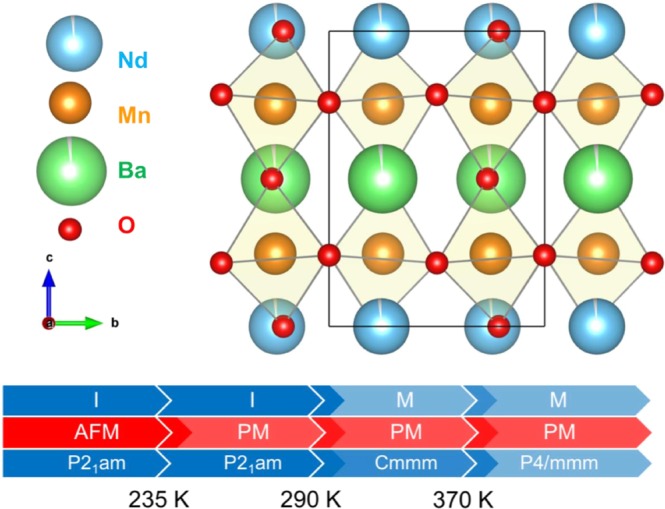
(**a**) Crystal structure of NdBaMn_2_O_6_ at low temperatures^28^. (**b**) Schematic depicting the electric, magnetic, and structural transitions occurring in NdBaMn_2_O_6_ (M: metallic, I: insulator, AFM: antiferromagnetic, and PM: paramagnetic).

A detailed understanding of the structural, magnetic, and transport properties of NdBaMn_2_O_6_ can be obtained by analyzing isotropic single-crystal^26,27^ and polycrystalline samples. At room temperature, single-crystal NdBaMn_2_O_6_ has an orthorhombic structure with a *Cmmm* space group, and the MnO_6_ octahedral exhibits no tilting. At temperatures higher than 370 K, NdBaMn_2_O_6_ has a symmetric *P4/mmm* structure, which is consistent with polycrystalline samples. At temperatures lower than 300 K, MnO_6_ octahedra alternately tilt by approximately 5° around the *a*-axis because of the contraction of the *ab*-plane and elongation of the *c-*axis, which coincide with the charge/orbital ordering. This structural transition coupled with the orbital-order transition result in a three-order change in the resistivity of the metal–insulator transition at 290 K. The octahedral tilt increases up to 10° with cooling, which modifies the structure to include the orthorhombic space group *P2*_1_*am*. The direction of the octahedral tilt at low temperatures is 45° from that at 300 K^26^. Temperature-dependent magnetic susceptibility measurements revealed that the material became antiferromagnetic below *T*_N_ = 235 K. The crystal structures and phase transitions are summarized in Fig. 1 ^28^.

Despite vast research conducted on NdBaMn_2_O_6_, its optical and vibrational properties have remained largely unexplored^21–27^. In this study, we used spectroscopic ellipsometry and Raman scattering spectroscopy to explore the electronic structure and lattice dynamics of NdBaMn_2_O_6_. Understanding the intrinsic mechanisms governing the optical and phononic excitations is important for device applications. We also studied the correlation between the temperature-dependent optical response and complex phase transitions of NdBaMn_2_O_6_, which helped in elucidating the nature of interactions among the charge, structure, and magnetism in this system and provided a strategy for controlling the functionality of double perovskite manganites.

## Experiment

The floating zone method was used to grow single crystals of NdBaMn_2_O_6_. The details of sample preparation and characterization are provided in^26^. The crystals with the (001) surface used in this study had approximate dimensions of 3 × 3 × 0.5 mm^3^. The spectroscopic ellipsometry measurements were performed under angles of incidence between 60° and 75° using a Woollam M-2000U ellipsometer over a spectral range of 0.73–6.42 eV. For temperature dependent measurements between 4.5 and 500 K, the ellipsometer was equipped with a Janis ST-400 ultrahigh-vacuum cryostat. Due to the 70° angle of the two cryostat windows, only a single angle of incidence is possible. The Raman scattering spectra were measured in a backscattering configuration using a laser with an excitation wavelength of 532 nm and a SENTERRA spectrometer with a 1024-pixel-wide charge-coupled detector. The incident beam is parallel to the z-axis and was kept at 2.0 mW. The polarized Raman scattering spectra were obtained in backscattering geometry from four scattering configurations: Z(Y, Y)$\bar{Z}$, Z(Y, X)$\bar{Z}$, Z(Y′, Y′)$\bar{Z}$, and Z(Y′, X′)$\bar{Z}$. In this Porto notation, the first and the last letter represent the directions of the incident and the scattered light, whereas the letters in parentheses indicate the polarizations of the incident and scattered light, respectively. X, Y, and Z are parallel to the orthorhombic [100], [010], and [001] crystal directions while X′ and Y′ are along $[110]$ and $[\bar{1}10]$ crystal directions, respectively. The sample was placed in a continuous-flow helium cryostat and LINKAM heating stage, which allowed measurements in the temperature range of 20–500 K. Apart from marginal intensity changes, no significant differences were observed in the spectra obtained at 300 K between the low- and high-temperature set-ups.

## Results and Discussion

### Electronic excitation

**Figure 2:**
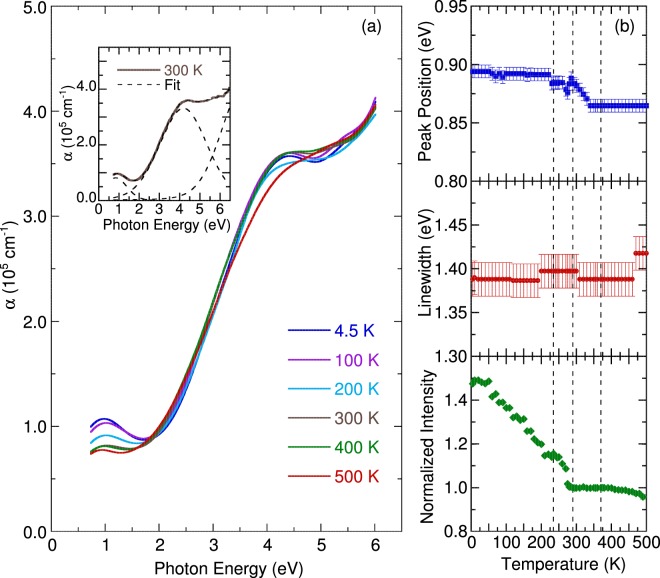
(**a**) Temperature dependence of the optical absorption spectra of NdBaMn_2_O_6_. The inset illustrates the best fit with the Lorentz-Gaussian function at 300 K. (**b**) The temperature dependence of the *d–d* excitation energy, linewidth and normalized intensity. The vertical dashed lines denote the transition temperatures.

The inset of Fig. 2(a) displays the room temperature optical absorption spectrum of NdBaMn_2_O_6_ obtained through spectroscopic ellipsometry. The room temperature spectrum were fitted using a Lorentz-Gaussian function, which accounts the broadening at room temperature. The absorption was resolved into two peaks at approximately 0.88 and 4.10 eV. We did not focus on the third band, because its peak exceeded the measured photon energy range. The origin of these peaks was explained using different mechanisms because of the complicated degrees of freedom associated with perovskite manganites. Noh *et al*.^29^ proposed a model comprising peaks at 1.5 (associated with the interorbital transition in the same Mn^3+^ site) and 4.5 eV (associated with the transition between the 2*p* state of O and the 3*d* state of Mn). This model is based on double exchange and phonon–electron interaction. Accordingly, the peak at 0.88 eV can be assigned to *d–d* excitation. This is similar with the peak observed for SmBaMn_2_O_6_ at 1.1 eV which was also assigned to *d–d* excitation^21^. Although SmBaMn_2_O_6_ and NdBaMn_2_O_6_ are structurally close compounds, the small difference in their size results to large differences in their ground state properties. At room temperature NdBaMn_2_O_6_ shows metallic properties while SmBaMn_2_O_6_ is an insulator. Thus, the discrepancy in the observed peak can be explained by the Jahn-Teller effect. It was pointed out that the smaller Sm ions are expected to have a larger mismatch and greater Jahn-Teller distortions resulting to a higher energy peak compared to NdBaMn_2_O_6_. Previous study on Nd_0.7_Sr_0.3_MnO_3_^30^ reported a broad peak at 1.2 eV. This peak was assigned to the charge transfer from Mn^3+^ to Mn^4+^, which was most likely caused by the Jahn–Teller effect coupled with the exchange phenomena. Other studies have also reported the same results^2,29–33^. At the higher energy range between 2.0 and 5.0 eV, the optical spectrum of Nd_0.7_Sr_0.3_MnO_3_ at 300 K resembles that of NdBaMn_2_O_6_. Nd_0.7_Sr_0.3_MnO_3_ features a peak at 4.5 eV due to charge transfer transitions between the O *2p* and Mn *e*_*g*_ bands^30^. Parallel to this, the peak at 4.10 eV was assigned to charge-transfer transitions between the 2*p* state of O and 3*d* state of Mn, which is consistent with other perovskite manganites^2,29–33^. The higher energy bands are well predicted by the ionic model and a common feature in RMnO_3_ systems^34,35^. Insufficient data at energies beyond 2.0 eV for SmBaMn_2_O_6_ limits the comparison.

Figure 2(a) illustrates the temperature dependence of the optical absorption spectra. As the temperature decreased, all optical absorptions exhibited shifts of the peak positions to higher energies and narrowing of linewidths. Figure 2(b) denotes the peak energy, linewidth, and normalized intensity of 0.88 eV *d-d* excitation as a function of temperature. Notably, the *d–d* excitation energy shows anomalies at 290 and 235 K, accompanied by an enhanced intensity below 290 K. As previously mentioned, below 290 K the charge/orbital ordering develops and NdBaMn_2_O_6_ exhibits an insulating behavior^24,26,27^ as the MnO_6_ octahedra tilting. The anomalies in energy and intensity are a reflection of how the *d–d* excitation band captures electrons in the conduction band resulting to a lower conductivity upon cooling. These anomalies below 290 K also emphasize the role of the octahedra tilting on the metal-insulator transition. In addition, the *d–d* excitation shows discontinuity in the energy and intensity near the 235 K Neel temperature, implying that a change in the electronic structure of NdBaMn_2_O_6_ occurs due to the effect of long-range antiferromagnetic ordering. This behavior suggests a strong coupling between the electronic structure and magnetic ordering through charge-spin interactions.

### Vibrational properties

**Figure 3:**
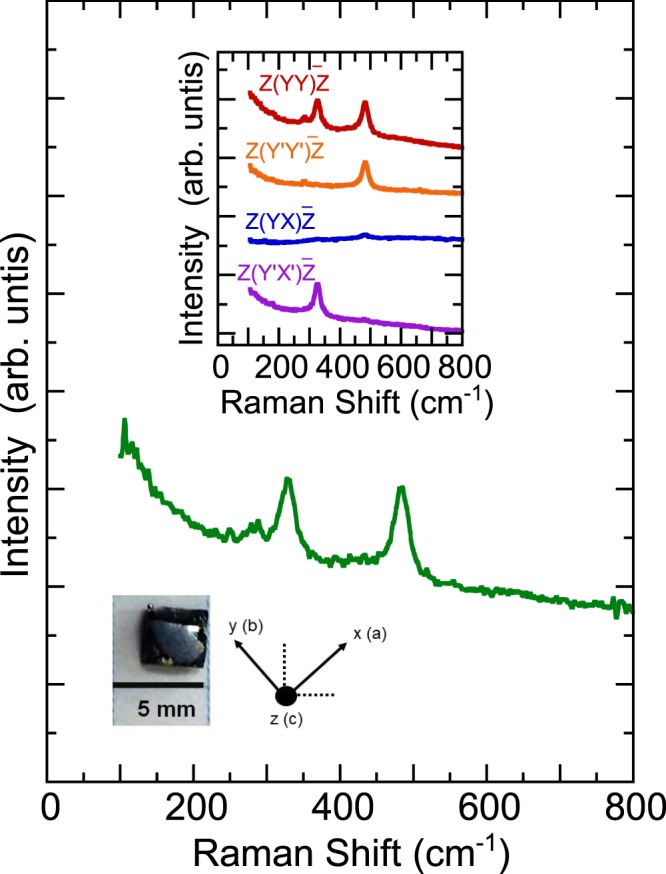
Unpolarized room-temperature Raman scattering spectrum of NdBaMn_2_O_6_. The inset illustrates the polarized Raman scattering spectra and the optical image of NdBaMn_2_O_6_ single crystal. The notations used for the crystallographic directions are also given.

Figure 3 displays the room-temperature Raman scattering spectrum of NdBaMn_2_O_6_. The spectrum comprises three first-order Raman phonon modes. We fitted these phonon peaks using a standard Lorentzian profile. According to factor group analysis^36^, NdBaMn_2_O_6_ crystallizes with a *Cmmm* orthorhombic structure at 300 K. This structure contains four molecules in the Bravais cell (*Z* = 4) located in the 4 *g* (Nd), 4 *h* (Ba), 8*n* (Mn), 4*i* (O), 4*j* (O), 4*k* (O), 4*l* (O), and 8 m (O) Wyckoff sites. The motion of the Nd and Ba atoms is represented as ${\varGamma }_{Nd}={\varGamma }_{Ba}={A}_{g}+{B}_{3u}+{B}_{1g}+{B}_{2u}+{B}_{2g}+{B}_{1u}$. The motion of the Mn atoms can be represented as ${\varGamma }_{Mn}=2{A}_{g}+2{B}_{3g}+{A}_{u}+{B}_{3u}+\,{B}_{1g}+{B}_{2g}+2{B}_{1u}+2{B}_{2u}$. Adding the contribution from the O atoms, the irreducible representation of the phonon modes at the center of the Brillouin zone is ${\varGamma }_{vib}=9{A}_{g}+6{B}_{1g}+\,7{B}_{2g}+8{B}_{3g}+2{A}_{u}+8{B}_{1u}\,+\,9{B}_{2u}\,+8{B}_{3u}$. The ${A}_{g},{B}_{1g},\,{B}_{2g},\,\,{\rm{and}}\,{B}_{3g}$ modes are Raman active; *B*_1*u*_, *B*_2*u*_ and *B*_3*u*_ modes are infrared active; and *A*_*u*_ mode is neither Raman nor infrared active. The NdBaMn_2_O_6_ structure with the *Cmmm* space group includes 30 Raman-active modes and only 9*A*_*g*_ and 6*B*_1*g*_ modes are expected considering the crystal orientation adapted. Below 290 K the crystal structure shifts to *P2*_*1*_*am*. The phonon modes for this structure is given by ${\varGamma }_{vib}=16{A}_{1}+13{A}_{2}+\,12{B}_{1}+16{B}_{2}.$ The *A*_1_, *B*_1_ and *B*_2_ are Raman active with 16*A*_1_ modes expected to be observed in our adapted orientation. At temperatures higher than 370 K, the NdBaMn_2_O_6_ crystal transitions from an orthorhombic *Cmmm* structure to a tetragonal *P4/mmm* structure. There are 18 Γ-point phonon modes for this structure composed of $2{A}_{1g}+{B}_{1g}+5{A}_{2u}+{B}_{2u}+3{E}_{g}+\,6{E}_{u}.$ The *A*_1*g*_, *B*_1*g*_, and *E*_*g*_ modes are Raman active which gives a total of six modes. The *A*_2*u*_ and *E*_*u*_ modes are infrared active, whereas *B*_2*u*_ is a silent acoustic mode. Out of the six Raman-active modes, only three modes composed of two *A*_1*g*_ and one *B*_1*g*_ are expected in the *P4/mmm* considering the crystal orientation employed in the study. Additional details of the factor group analysis are provided in the supplementary information.

**Figure 4:**
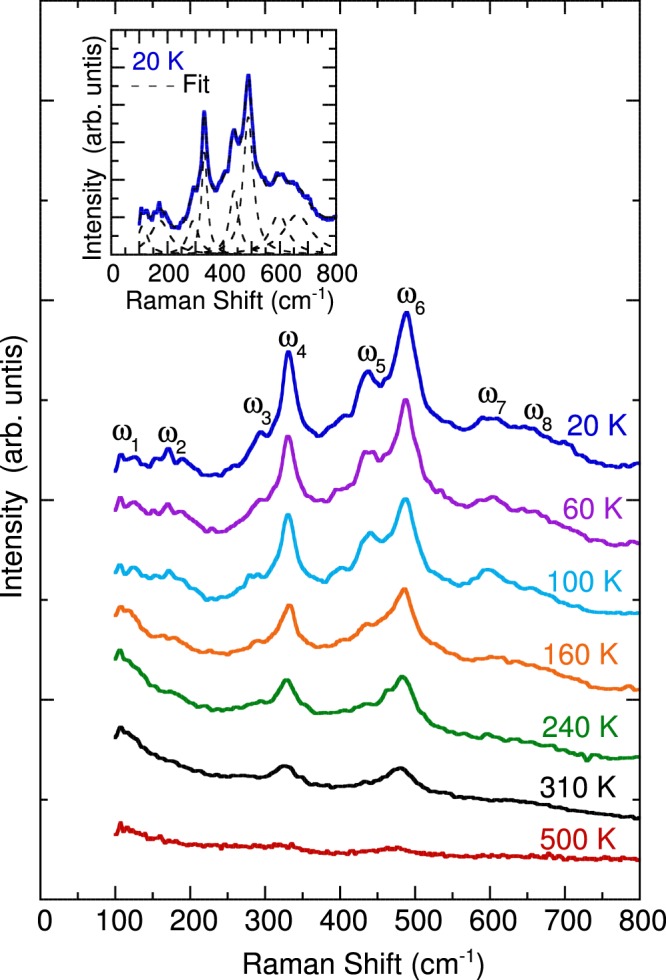
Temperature dependence of the unpolarized Raman scattering spectra of NdBaMn_2_O_6_. The inset illustrates the results of fitting the spectrum obtained at 20 K by using the Lorentzian model. The background was removed from the linear fits described in the text.

We observed a total of three Raman-active phonon modes at room temperature. This is consistent with the expected phonon modes for *P4/mmm*. The peaks are composed of two intense modes at approximately 320 and 480 cm^−1^ and a small peak around 270 cm^−1^. The inset of Fig. 3 displays the polarized Raman scattering spectra in four different configurations. The modes at 270, 320, and 480 cm^−1^ exhibited a higher intensity in the parallel configuration (YY) than in the cross configuration (YX). This satisfies the selection rule for *P4/mmm* in which *A*_1*g*_ and *B*_1*g*_ modes should appear in the YY configuration and no peaks are expected for the YX configuration. On the other hand, the primed spectra Y′Y′ and Y′X′ are different compared to its unprimed counterpart YY and YX which shows that the sample is a single crystal. The Y′Y′ spectrum shows the presence of the mode around 480 cm^−1^ and the weak peak around 270 cm^−1^. The Y′X′ spectrum shows the suppressed intensity of the 270 and 480 cm^−1^ phonon modes while exhibiting a strong intensity of phonon mode at 320 cm^−1^. We can deduce that the 270 and 480 cm^−1^ phonon modes have *A*_1*g*_ symmetry while the 330 cm^−1^ phonon mode has *B*_1*g*_ symmetry. The 270 cm^−1^ mode was assigned to the octahedral rotation mode and can be used as a measure of octahedral tilting^37^. The 320 cm^−1^ mode was assigned to MnO_6_ octahedral out-of-phase tilting and bending. The 480 cm^−1^ mode was assigned to the Jahn–Teller distortion, which mainly involves symmetric stretching of O atoms. These assignments were parallel to SmBaMn_2_O_6_^21^ and other perovskite materials^37–41^. The temperature-dependent Raman scattering spectra of NdBaMn_2_O_6_ are displayed in Fig. 4. The intensity of the phonon modes weakened when the temperature was increased to 500 K. By contrast, the Raman scattering spectrum exhibited sharp phonon modes at 20 K. Eight Lorentzian oscillators were used to represent the Raman scattering spectrum at 20 K (inset of Fig. 4), whereas the background was taken to be linear in these fits using the form *A*ω + *B*, where *A* and *B* are adjustable parameters. The mode frequency and assignment are summarized in Table 1.

**Table 1:** Raman peaks observed at 20 K with their corresponding symmetry assignments.

Frequency (cm^−1^)			
*ω*_1_	117	Mixed vibrations from Nd and Ba	
*ω*_2_	177		
*ω*_3_	296	Octahedral distortion	Out-of-phase rotation
*ω*_4_	332		Tilting/bending
*ω*_5_	442	Jahn-Teller stretching	
*ω*_6_	489		
*ω*_7_	600	Breathing modes	
*ω*_8_	653		

Raman scattering spectra of half-doped manganites Nd_0.5_Sr_0.5_MnO_3_^42^ and La_0.5_Ca_0.5_MnO_3_^43^ can be associated with that of NdBaMn_2_O_6_ which are summarized in Table 2. At 280 K, Raman peaks for Nd_0.5_Sr_0.5_MnO_3_ are centered around 205, 415, and 444 cm^−1^ with 335, 489, and 610 cm^−1^ appearing as temperature was lowered. These peaks also have a counterpart for La_0.5_Ca_0.5_MnO_3_ at 337, 473, 487 and 601 cm^−1^. At the same temperature, NdBaMn_2_O_6_ shows similar peaks at 272, 327, 444 and 483 cm^−1^. The 327 cm^−1^ mode for NdBaMn_2_O_6_ which describes the distortion of the octahedra corresponds to 335 and 337 cm^−1^ modes for Nd_0.5_Sr_0.5_MnO_3_ and La_0.5_Ca_0.5_MnO_3_, respectively. The 444 and 483 cm^−1^ Jahn-Teller modes corresponds to 415 and 444 cm^−1^ modes for Nd_0.5_Sr_0.5_MnO_3_ and 473 and 487 cm^−1^ modes for La_0.5_Ca_0.5_MnO_3_. Similar with Nd_0.5_Sr_0.5_MnO_3_, the breathing mode of NdBaMn_2_O_6_ at 612 cm^−1^ also appears when temperature was lowered. The higher frequency breathing mode at 653 cm^−1^ for NdBaMn_2_O_6_ was also observed for the half-doped manganites when temperature was lowered. The 205 cm^−1^ mode for Nd_0.5_Sr_0.5_MnO_3_ (230 cm^−1^ mode for La_0.5_Ca_0.5_MnO_3_) which is associated with the manganite rotation-like mode and also a measure of the average angle of octahedral tilt^43,44^ corresponds to 272 cm^−1^ mode for NdBaMn_2_O_6_. This peak appears relatively weak compared to Nd_0.5_Sr_0.5_MnO_3_ and La_0.5_Ca_0.5_MnO_3_. This might be due to the layered structure in the A-site ordering. A dramatic enhancement in intensity below 235 K was observed for this peak, indicating the increase in the tilting of the MnO_6_ as temperature decreases. Although Raman peaks of half-doped manganites and the A-site ordered manganites have a strong similarity, a number of relatively weak peaks that appear in both Nd_0.5_Sr_0.5_MnO_3_ and La_0.5_Ca_0.5_MnO_3_ cannot be accounted in NdBaMn_2_O_6_. These peaks are centered at 316, 358, 509, 216, 258, 401, and 428 cm^−1^ in Nd_0.5_Sr_0.5_MnO_3_^42^ with corresponding peaks at 319, 359, 516, 217, 270, 401, and 429 cm^−1^ in La_0.5_Ca_0.5_MnO_3_^43^. The appearance of these peaks in Nd_0.5_Sr_0.5_MnO_3_ and La_0.5_Ca_0.5_MnO_3_ agrees with previous studies^42,43,45^ that these peaks originates from the charge ordering. NdBaMn_2_O_6_ exhibits charge/orbital ordering reaching up to room temperatures which competes with ferromagnetic interaction^24^. The charge/orbital ordering melts above room temperature upon the enhancement of the A-site ordering. Based on these comparisons with the half-doped disordered manganites, we can associate A-site ordering signature to less random tilting modes characterize by fewer and distinct Raman peaks below 300 cm^−1^. Additionally, A-site ordering results to narrower phonon linewidths compared to those of disordered manganites^42–46^.

**Table 2:** Raman peaks observed at 280 K for NdBaMn_2_O_6_ with the corresponding peaks for SmBaMn_2_O_6_, Nd_0.5_Sr_0.5_MnO_3_, and La_0.5_Ca_0.5_MnO_3_. Frequencies labelled with (A) are modes that appear at low temperature.

Mode	Frequency (cm^−1^)			
	NdBaMn_2_O_6_	SmBaMn_2_O_6_^21^	Nd_0.5_Sr_0.5_MnO_3_^42^	La_0.5_Ca_0.5_MnO_3_^43^
Mixed mode Octahedral distortion (tilting/bending/rotation)	272 327		205 335 (A)	230 337
Jahn-Teller stretching	444 (A) 483	496	415 444 489 (A)	473 487
Breathing modes	612 (A) 653 (A)	620	610 (A) 651 (A)	601 643 (A)

It would also be interesting to compare the counterpart of the peaks in NdBaMn_2_O_6_ to SmBaMn_2_O_6_ especially the octahedra tilting mode. Previous study on the Raman scattering spectra of manganites with general formula RMnO_3_ reveals that this mode shifts to higher frequency as the ionic radius of the rare earth element decreases^47^. However, the Raman data available in literature is limited only between 350 and 750 cm^−1^. Still, similar spectral lines observed for SmBaMn_2_O_6_ at 500 cm^−1^ Jahn-Teller peak has counterpart in NdBaMn_2_O_6_ at 490 cm^−1^ while the breathing mode peak at 620 cm^−1^ was resolved into two peaks at 600 and 653 cm^−1^. The slight shift in the frequency in these neighboring compounds can be ascribed to the influence of the bond angle changes brought about by their difference in size.

**Figure 5:**
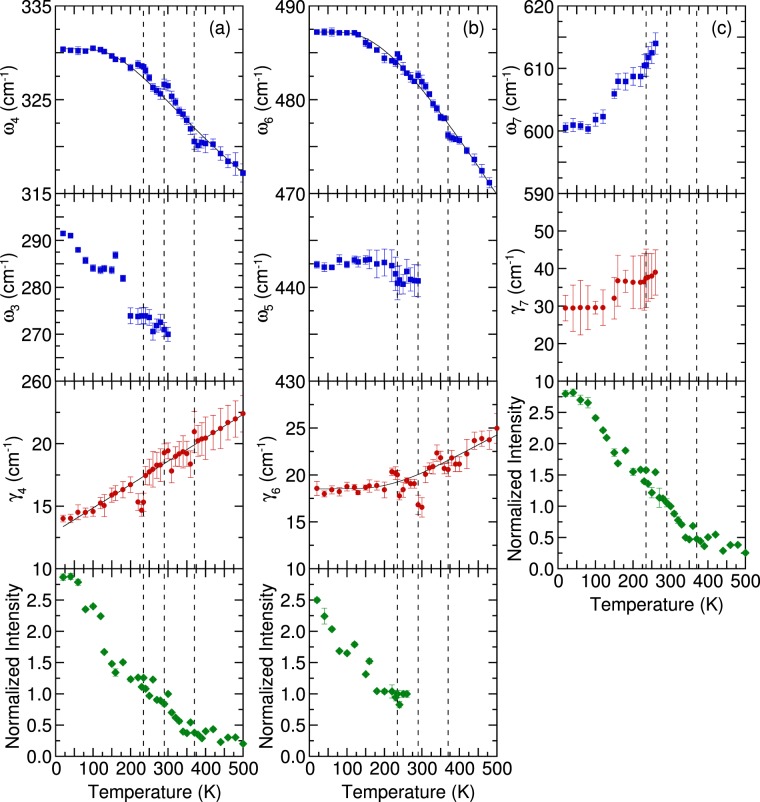
Temperature dependence of the frequency, linewidth, and normalized intensity of the phonon modes. The thin solid lines are the fitting results obtained with the anharmonic model. The vertical dashed lines denote the transition temperatures.

When the temperature decreased, the peak positions of most of the phonon modes of NdBaMn_2_O_6_ shifted to higher frequencies, and their resonance linewidth decreased. However, the phonon parameters of the octahedral bending (320 cm^−1^) and Jahn–Teller (480 cm^−1^) modes exhibited peculiar behavior at 370, 290, and 235 K. A shoulder peak began to appear alongside the strong 480 cm^−1^ mode when the temperature was reduced to less than 290 K while the 270 cm^−1^ mode shows a dramatic enhancement. Moreover, two breathing modes with frequencies higher than 600 cm^−1^ appeared below the magnetic transition temperature (*T*_N_ = 235 K). Figure 5 illustrates the peak frequency, linewidth, and normalized intensity of the phonon modes as functions of the temperature. The frequencies and linewidths of the octahedral bending (320 cm^−1^) and Jahn–Teller (480 cm^−1^) modes changed discontinuously at 370, 290, and 235 K. In a normal anharmonic solid, the oscillator strength of the phonon mode is expected to be independent of the temperature^39,48^. Moreover, at decreasing temperature, the phonon frequency should increase and the linewidth should decrease. The anharmonic dependence of the phonon frequency and linewidth is expressed as follows:

1 $$\omega \,(T)={\omega }_{0}+A(1+\frac{2}{\exp (\frac{\Theta }{T})-1})$$

2 $$\gamma (T)={\gamma }_{0}+B(1+\frac{2}{\exp (\frac{\Theta }{T})-1})$$

where *ω*_0_ is the intrinsic frequency of the optical phonon mode, *γ*_0_ is the linewidth broadening caused by defects, Θ is the Debye temperature, and *A* and *B* are the anharmonic coefficients^49^. This model was used to get the best fit of the frequency and linewidth for temperatures above the magnetic ordering (235 K). For the octahedral bending mode at 327 cm^−1^, the following values were obtained: *ω*_0_ ≈ 332.1 cm^−1^, *γ*_0_ ≈ 9.3 cm^−1^, *A* ≈ −9 cm^−1^, and *B* ≈ 5 cm^−1^. For the Jahn–Teller mode at 482 cm^−1^, the following values were obtained: *ω*_0_ ≈ 497 cm^−1^, *γ*_0_ ≈ 18.7 cm^−1^, *A* ≈ −10.7 cm^−1^, and *B* ≈ 7.6 cm^−1^. The average Debye temperature was 478 K. The negative sign for *A* indicated that the phonon frequency increased as temperature decreased. The positive sign for *B* indicated that the linewidth decreased with a decrease in the temperature. The thin solid lines in Fig. 5 represent the theoretical predictions based on Eqs. (1) and (2) which describes the best fit line of the frequencies and linewidth above the magnetic ordering temperature (235 K). The octahedral bending mode at 327 cm^−1^ and the Jahn–Teller mode at 482 cm^−1^ deviated from the usual anharmonic contribution to the temperature dependence of the phonon frequency and linewidth because of the structural phase transition at 370 K, metal–insulator transition at 290 K, and antiferromagnetic ordering transition at 235 K. The deviation observed in the high-temperature phase could be attributed to the structural phase transition from tetragonal to orthorhombic symmetry at 370 K, anomalies in the lattice constant, and an increase in the Jahn–Teller distortions at 290 K^26^. However, NdBaMn_2_O_6_ exhibited no drastic changes in its crystal structure and lattice constants in the low-temperature phase. The phonon anomalies at 235 K were most likely caused by spin–phonon interactions. The shoulder phonon peak appeared at approximately 440 cm^−1^ together with the enhancement of the 270 cm^−1^ phonon peak for a temperature lower than the metal–insulator transition temperature of 290 K. These phonon modes were likely activated by orthorhombic distortion because the *Cmmm* structure was modified to the *P2*_*1*_*am* structure, which has lower orthorhombic symmetry. These phonon behaviors were directly correlated with the onset of the charge/orbital ordering, which was observed in NdBaMn_2_O_6_ at 290 K^25^. The rotational distortion (270 cm^−1^) accompanied with the Jahn-Teller octahedral distortions (440 cm^−1^) was accounted to induce this ordering^13^.

**Figure 6:**
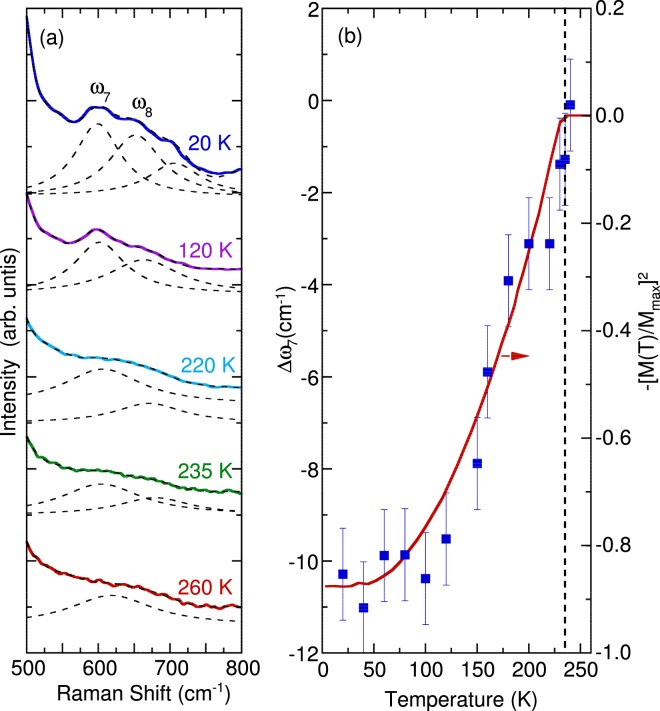
(**a**) Temperature dependence of the Raman scattering spectra between 500 and 800 cm^−1^. The dashed lines indicate the best fit with the Lorentzian model. (**b**) Temperature dependence of the shift in the phonon frequency of the breathing mode (with respect to its value at 260 K) plotted against the normalized square of the magnetic susceptibility^26^.

The temperature evolution of the breathing mode at approximately 610 cm^−1^ is illustrated in Fig. 6. A third breathing mode at approximately 700 cm^−1^ was added in fitting from 20 K up to 60 K to accurately demonstrate the normal anharmonic linewidth behavior of the other breathing modes. We do not focus on this phonon mode because of its very weak intensity. The phonon associated with completely symmetrical oxygen stretching vibrations gradually red-shifted when the temperature decreased from 235 to 20 K. The other breathing mode near 660 cm^−1^ exhibited similar behavior. This softening is associated with the renormalization of the phonon induced by magnetic ordering, which is a signature of spin–phonon coupling. A similar softening for the 601 cm^−1^ was observed for NdMnO_3_ which also exhibit an *A* type antiferromagnetism at low temperature. By contrast, the 607 cm^−1^ mode in Nd_0.5_Sr_0.5_MnO_3_ which appears with the occurrence of the *CE* type antiferromagnetism temperatures shows a hardening to 610 cm^−1^ when temperature was lowered. No detailed analysis of the breathing mode was reported for SmBaMn_2_O_6_^10^ which also exhibits *CE* type antiferromagnetism. This behavior confirms the sensitivity of the spin-phonon coupling to the spin ordering^42,45,46^.

The observed phonon softening correlated well with the normalized square of magnetic susceptibility data measured for NdBaMn_2_O_6_^26^, as illustrated in Fig. 6(b). The spin–phonon contribution was caused by the modulation of the exchange integral through lattice vibrations, which is predicted in the mean field theory^39^. Thus, in a simple model, the shift in phonon frequency caused by magnetic ordering is given as follows:

3 $$\Delta \omega (T)\approx \lambda \langle {S}_{i}\cdot {S}_{j}\rangle \approx 6\lambda {(\frac{M(T)}{{M}_{s}})}^{2}$$

where Δ*ω*(*T*) is the shift in phonon frequency, *λ* is the spin–phonon coupling constant, and $\langle {S}_{i}\cdot {S}_{j}\rangle$is the nearest neighbor spin correlation. The $\langle {S}_{i}\cdot {S}_{j}\rangle$ can be estimated from $6{(\frac{M(T)}{{M}_{s}})}^{2}$where *M(T)* is the magnetization per magnetic ion, *M*_*s*_ is the saturation magnetization and a factor of 6 considering the number of nearest neighbor^39,46,50,51^. In the present study, we used the magnetic susceptibility with the external magnetic field along the *ab* plane. We estimated the renormalization of the phonon frequency due to the spin-phonon interaction by subtracting the anharmonic contributions, which can be estimated from the high temperature phonon frequencies. In this regard, we neglected the lattice effects and used the data at 260 K as the reference since the breathing mode becomes too weak and broad to be clearly resolved at higher temperature. Also, from the fitting results of the breathing modes at 260 K it can be observed that the phonon frequency still exhibits softening above the magnetic ordering temperature. This is caused by the in plane ferromagnetic correlations that exists in this temperature range^27^. In addition, Raman scattering spectra in the breathing modes region do not change significantly at the high temperature phase where structural phase transition occurs. These results further justifies that the spin-phonon interaction plays a more important role in the softening of this mode rather than the lattice contributions. From Eq. (3) and the data from Fig. 6, the spin–phonon coupling constant was estimated to be *λ* = 2.5 cm^−1^.

A quantitative value of *λ* can also be obtained using a simplified lattice model proposed by Sushkov *et al*.^52^ which is given as follows:

4 $$\lambda \approx \frac{2{\alpha }^{2}J}{m\omega }$$

where *m* is the mass of the magnetic ion, *ω* the mode frequency, *J* the nearest neighbor exchange coupling constant, and *α* can be calculated using *α* = 2*z*/3*a*_*B*_ (*a*_*B*_ is the Bohr radius and z is the nearest neighbor coordination number). Considering only the nearest neighbors (z = 6), we obtained $\alpha =7.56/{\rm{\AA }}.$ Meanwhile, we estimated the exchange coupling constant using

5 $$J=3{k}_{B}{\Theta }_{cw}/zS(S+1)$$

where *k*_*B*_ is the Boltzmann constant, ${\Theta }_{cw}$ is the Currie-Weiss temperature, and *S* is the spin angular moment^52,53^. Here, we used z = 6, *S* = 7/4 and ${\Theta }_{cw}=235\,K$ taken from ref. ^25^ to get *J* = 2.1 *meV*. Thus, we get the spin-phonon coupling constant *λ* ≈ 2 cm^−1^ which is close to our obtained value of *λ* = 2.5 cm^−1^. These values are comparable to perovskite manganites and magnetic oxide materials in other studies^39,52–56^.

## Summary

We used spectroscopic ellipsometry and Raman scattering spectroscopy to determine the electronic structure and lattice dynamics of NdBaMn_2_O_6_ single crystals. The room-temperature optical absorption spectrum of NdBaMn_2_O_6_ indicated the occurrence of *d*–*d* on-site transitions in Mn at approximately 0.88 eV. The spectrum also indicated the occurrence of charge-transfer transitions between the 2*p* state of O and 3*d* of Mn at approximately 4.10 eV. The temperature dependent peak energy and intensity of the *d*–*d* excitation presented anomalies at 290 and 235 K. The octahedral bending mode at 327 cm^−1^ and Jahn–Teller mode at 482 cm^−1^ indicated the sensitivity of the single crystals to the structural phase transition at 370 K, metal–insulator transition at 290 K, and antiferromagnetic ordering transition at 235 K. Moreover, the activated phonon mode, which appeared at approximately 440 cm^−1^ alongside with the enhancement of the 270 cm^−1^ phonon mode, coupled strongly to the metal–insulator transition at 290 K, which was associated with charge/orbital ordering. The temperature dependence of the breathing mode at 610 cm^−1^ exhibited an anomalous softening at a temperature less than 235 K. The spin–phonon coupling constant obtained was *λ* = 2.5 cm^−1^ which is close to the calculated value of *λ* ≈ 2 cm^−1^. These results confirmed a strong interaction among the charge, orbital, and spin degrees of freedom in NdBaMn_2_O_6_.

## Supplementary information

Supplementary information
