# Supplementary material for: Optical Study of the Electronic Structure and Lattice Dynamics of NdBaMn2O6 Single Crystals
Source: Sci Rep. 2019 Dec 3;9:18164. doi: 10.1038/s41598-019-54524-0 (PMC6890712; doi:10.1038/s41598-019-54524-0)
Supplement: Supplementary file 1 — Supplementary information [file 41598_2019_54524_MOESM1_ESM.pdf]

Supplementary information

Optical Study of the Electronic Structure and Lattice Dynamics of $NdBaMn_2O_6$ Single Crystals

Rea Divina Mero[1], Kirari Ogawa[2], Shigeki Yamada[2], and Hsiang-Lin Liu[1,*]

[1]*Department of Physics, National Taiwan Normal University, Taipei 11677, Taiwan*
[2]*Department of Material System Science, Yokohama City University, Yokohama 236-0027, Japan*

*Corresponding author: hliu@ntnu.edu.tw

Orthorhombic $P2_1am$ $NdBaMn_2O_6$

Atom	Wyckoff notation	Site symmetry	Irreducible representation
Nd	2a	C_s	$\mathbf{\Gamma_{Nd} = 2A_1 + A_2 + B_1 + 2B_2}$
Ba	2b	C_s	$\mathbf{\Gamma_{Ba} = 2A_1 + A_2 + B_1 + 2B_2}$
Mn	4c	C_{2v}	$\mathbf{\Gamma_{Mn} = 3A_1 + 3A_2 + 3B_1 + 3B_2}$
O1	2a	C_s	$\mathbf{\Gamma_o = 2A_1 + A_2 + B_1 + 2B_2}$
O2	2b		
O3	4c	C_{2v}	$\mathbf{\Gamma_o = 3A_1 + 3A_2 + 3B_1 + 3B_2}$
O4	4c		

$$\Gamma_{crystal} = 17A_1 + 13A_2 + 13B_1 + 17B_2$$

$$\Gamma_{acoustic} = A_1 + B_1 + B_2$$

$$\Gamma_{vib} = 16A_1(R, IR) + 13A_2(IR) + 12B_1(R\ , IR) + 16B_2(R\ , IR)\ )$$

R – Raman active
IR – Infrared active

Polarization selection rules for *P2₁am*					
	A_1(LO)	A_1(TO)	A_2	B_1(TO)	B_2(TO)
$X(YY)\overline{X}$		✓			
$X(YZ)\overline{X}$					✓
$X(ZZ)\overline{X}$		✓			
$Y(XX)\overline{Y}$		✓			
$Y(XZ)\overline{Y}$				✓	
$Y(ZZ)\overline{Y}$		✓			
$Z(XX)\overline{Z}$	✓				
$Z(XY)\overline{Z}$			✓		
$Z(YY)\overline{Z}$	✓				
$Z(X'X')\overline{Z}$	✓		✓		
$Z(X'Y')\overline{Z}$	✓				
$Z(Y'Y')\overline{Z}$	✓		✓		

Orthorhombic *Cmmm* $NdBaMn_2O_6$			
Atom	Wyckoff notation	Site symmetry	Irreducible representation
Nd	4g	$C_2(x)$	$\boldsymbol{\Gamma_{Nd} = A_g + B_{3u} + B_{1g} + B_{2u} + B_{2g} + B_{1u}}$
Ba	4h	$C_2(x)$	$\boldsymbol{\Gamma_{Ba} = A_g + B_{3u} + B_{1g} + B_{2u} + B_{2g} + B_{1u}}$
Mn	8n	C_s	$\boldsymbol{\Gamma_{Mn} = 2A_g + 2B_{3g} + A_u + B_{3u} + B_{1g} + B_{2}}$[illegible] $\boldsymbol{+ 2B_{1u} + 2B_{2u}}$
O1	4i	$C_2(y)$	$\boldsymbol{\Gamma_o = 2A_g + 2B_{3g} + 2B_{3u} + 2B_{1g} + 2B_{1u} + 2B_{2u}}$
O2	4j		
O3	4k	$C_2(z)$	$\boldsymbol{\Gamma_o = 2A_g + 2B_{3g} + 2B_{3u} + 2B_{2g} + 2B_{1u} + 2B_{2u}}$
O4	4l		
O5	8m		

$$\boldsymbol{\Gamma_{crystal} = 9A_g + 6B_{1g} + 7B_{2g} + 8B_{3g} + 2A_u + 9B_{1u} + 10B_{2u} + 9B_{3u}}$$

$$\boldsymbol{\Gamma_{acoustic} = B_{1u} + B_{2u} + B_{3u}}$$

$$\boldsymbol{\Gamma_{vib} = 9A_g\,(R) + 6B_{1g}(R) + 7B_{2g}(R) + 8B_{3g}(R) + 2A_u(IR) + 8B_{1u}(IR) + 9B_{2u}\,(IR) + 8B_{3u}(IR)}$$

R – Raman active
IR – Infrared active

Polarization selection rules for *Cmmm*				
	$\boldsymbol{A_g}$	$\boldsymbol{B_{1g}}$	$\boldsymbol{B_{2g}}$	$\boldsymbol{B_{3g}}$
$X(YY)\overline{X}$	✓			
$X(YZ)\overline{X}$				✓
$X(ZZ)\overline{X}$	✓			
$Y(XX)\overline{Y}$	✓			
$Y(XZ)\overline{Y}$			✓	
$Y(ZZ)\overline{Y}$	✓			
$Z(XX)\overline{Z}$	✓			
$Z(XY)\overline{Z}$		✓		
$Z(YY)\overline{Z}$	✓			
$Z(X'X')\overline{Z}$	✓	✓		
$Z(X'Y')\overline{Z}$	✓			
$Z(Y'Y')\overline{Z}$	✓	✓		

Tetragonal *P4/mmm* $NdBaMn_2O_6$			
Atom	Wyckoff notation	Site symmetry	Irreducible representation
Nd	1a	D_{4h}	$\boldsymbol{\Gamma_{Nd} = A_{2u} + E_u}$
Ba	1b	D_{4h}	$\boldsymbol{\Gamma_{Ba} = A_{2u} + E_u}$
Mn	2h	C_{4v}	$\boldsymbol{\Gamma_{Mn} = A_{1g} + +A_u + E_g + E_u}$
O1	1c	D_{4h}	$\boldsymbol{\Gamma_o = 2A_{2u} + 2E_u}$
O2	1d		
O3	4i	C_{2v}	$\boldsymbol{\Gamma_o = A_{1g} + B_{1g} + A_{2u} + B_{2u} + 2E_g + 2E_u}$

$$\boldsymbol{\Gamma_{crystal} = 2A_{1g} + B_{1g} + 6A_{2u} + B_{2u} + 3E_g + 7E_u}$$

$$\boldsymbol{\Gamma_{acoustic} = A_{2u} + E_u}$$

$$\boldsymbol{\Gamma_{vib} = 2A_{1g}(R) + B_{1g}(R) + 5A_{2u}(IR) + B_{2u}(IR) + 3E_g(R) + 6E_u\,(IR)}$$

R – Raman active
IR – Infrared active

Polarization selection rules for *P4/mmm*				
	$\boldsymbol{A_{1g}}$	$\boldsymbol{B_{1g}}$	$\boldsymbol{B_{2g}}$	$\boldsymbol{E_g}$
$X(YY)\overline{X}$	✓	✓		
$X(YZ)\overline{X}$				✓
$X(ZZ)\overline{X}$	✓			
$Y(XX)\overline{Y}$	✓	✓		
$Y(XZ)\overline{Y}$				✓
$Y(ZZ)\overline{Y}$	✓			
$Z(XX)\overline{Z}$	✓	✓		
$Z(XY)\overline{Z}$			✓	
$Z(YY)\overline{Z}$	✓	✓		
$Z(X'X')\overline{Z}$	✓		✓	
$Z(X'Y')\overline{Z}$		✓		
$Z(Y'Y')\overline{Z}$	✓		✓	
